# Development process of a mobile electronic medical record for nurses: a single case study

**DOI:** 10.1186/s12911-018-0726-3

**Published:** 2019-01-14

**Authors:** Danielle M. Vossebeld, Erik C. N. Puik, Joris E. N. Jaspers, Marieke J. Schuurmans

**Affiliations:** 1 Research Centre for Healthy and Sustainable Living, HU University of Applied Sciences Utrecht, P.O. Box 182, 3500 AD Utrecht, The Netherlands; 2 Department of Medical Technology & Clinical Physics, University Medical Center Utrecht, P.O. Box 85500, 3508 GA Utrecht, The Netherlands; 3 Julius Center, University Medical Center Utrecht, P.O. Box 85500, 3508 GA Utrecht, The Netherlands

**Keywords:** Mobile EMR, mHealth, Health information system, Mobile application, Axiomatic design, Product development process, Nurses, Customer needs

## Abstract

**Background:**

With the growing shortage of nurses, labor-saving technology has become more important. In health care practice, however, the fit with innovations is not easy. The aim of this study is to analyze the development of a mobile input device for electronic medical records (MEMR), a potentially labor-saving application supported by nurses, that failed to meet the needs of nurses after development.

**Method:**

In a case study, we used an axiomatic design framework as an evaluation tool to visualize the mismatches between customer needs and the design parameters of the MEMR, and trace these mismatches back to (preliminary) decisions in the development process. We applied a mixed-method research design that consisted of analyzing of 118 external and internal files and working documents, 29 interviews and shorter inquiries, a user test, and an observation of use. By factoring and grouping the findings, we analyzed the relevant categories of mismatches.

**Results:**

The involvement of nurses during the development was extensive, but not all feedback was, or could not be, used effectively to improve the MEMR. The mismatches with the most impact were found to be: (1) suboptimal supportive technology, (2) limited functionality of the app and input device, and (3) disruption of nurses’ workflow. Most mismatches were known by the IT department when the MEMR was offered to the units as a product. Development of the MEMR came to a halt because of limited use.

**Conclusion:**

Choices for design parameters, made during the development of labor-saving technology for nurses, may conflict with the customer needs of nurses. Even though the causes of mismatches were mentioned by the IT department, the nurse managers acquired the MEMR based on the idea behind the app. The effects of the chosen design parameters should not only be compared to the customer needs, but also be assessed with nurses and nurse managers for the expected effect on the workflow.

**Electronic supplementary material:**

The online version of this article (10.1186/s12911-018-0726-3) contains supplementary material, which is available to authorized users.

## Background

In 2012, a nurse and nurse manager participated in an innovation challenge with the idea of using a mobile application (app) for sending patient measurements directly at bedside to the electronic medical record (EMR) used at a University Medical Center in the Netherlands. At that time, measurements were initially written on paper and, at a later stage of the work shift, entered in the EMR. The expected benefit of this direct entry in the EMR via an app was, primarily, a more efficient work process, allowing more time for the patient, and increasing the quality of care [1].

The idea was adopted by the IT department. After two years of in-house development, the mobile EMR (MEMR) app was made available to the nursing units at the University Medical Center. The MEMR consisted of an app that ran on a standard mobile platform. Twelve nursing units acquired the MEMR. Within months the reactions varied from (partial) satisfaction to rejection. A nurse manager said: “we want that app, but not like this.” There appeared to be a mismatch between the nurses’ needs and the MEMR as it was implemented.

This specific mismatch is interesting. The idea for the MEMR app came from a staff member and was drawn up in co-operation with nurses; the development was supported by nurses and the app was tested by nurses. The question is: why was this app rejected after a short use? More specifically:

1. What were the factors contributing to this mismatch between nurses’ needs and the realized MEMR app?
2. At what point in the development process did this mismatch occur?

The goal of this research is to gain insight into how to design better products for and with nurses. The development of better applications can contribute to a more efficient work process, which is needed to help address the growing shortage of nurses [2, 3].

### Applications

The use of EMRs in hospitals is becoming more established in the Netherlands [4]. The use of mobile applications for healthcare professionals in hospitals is also emerging, as is scholarly knowledge on the factors influencing the adoption of these apps. To identify these factors, Lu [5] and Gagnon [6, 7] have reviewed articles on the adoption and use of handheld computers and apps. Gagnon [6, 7] groups the findings based on factors related to: (1) ICT, such as design and systems reliability, (2) the healthcare professional, such as attitudes towards ICT, and socio-demographic characteristics, (3) the human environment, with a focus on peers and patients, and (4) the organizational environment, including factors associated with work. The factors identified by Lu [5] also fit into these categories.

For most people, an app is more than mere software on a device [8]; it should be considered as part of a bigger system. Benyon [9] states: “People use technologies to undertake activities in contexts.” Therefore, to be able to design interactive systems, it is important to analyze the settings in which they are used. This entails taking account of not only the physical environment, but the social and organizational context too [9]. Indeed, Powell-Cope et al. [10] report that not all solutions will be beneficial if they are not properly designed for the user and context. Gould [11] similarly emphasizes the importance of a continuous focus on users and the importance of testing during the iterative process. Chang [12] also highlights the importance of involving nurses in designing effective and useful apps, and the needs of other stakeholders should also be taken into consideration when developing a product for nurses.

### Models

For an indication of the acceptance of IT solutions, the Technology Acceptance Model (TAM) or Unified Theory of Acceptance and Use of Technology (UTAUT) can be informative [13, 14]. Kuo [15] researched the adoption of MEMR systems using TAM, suggesting that a full understanding of the nurses’ requirements is crucial for designing a MEMR that meets the nurses’ needs. Both models are used to research the adoption of a MEMR by Kim [16], who mentions the importance of thoughtful analysis of user needs in order to comprehend the performance expectancy. In UTAUT, the performance expectancy is how the user perceives the usefulness and the beneficial effects of the technology on his/her work [17].

**Fig. 1:**
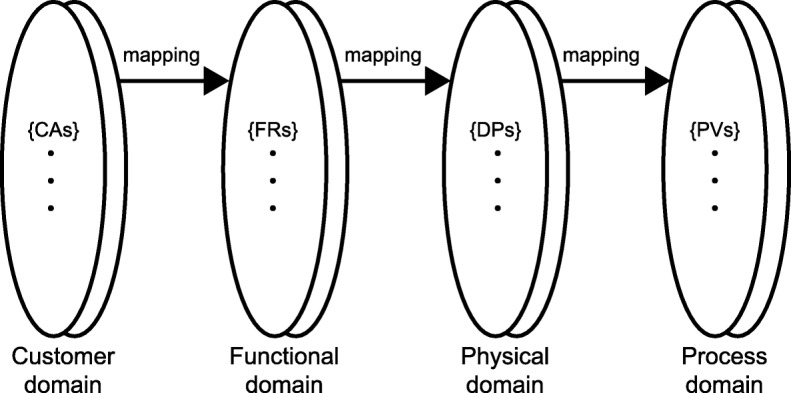
The domains of axiomatic design by Suh [20]

In the context of the present study, the perceived usefulness of the MEMR is based on the realized product compared to the requested functionality or expressed customer needs of the nurses. The actual use is based on their experience with the realized product, described by its design parameters (DPs). This can also be visualized with the domains of axiomatic design [18–20] (see Fig. 1).

Figure 1 illustrates Suh’s [20] model of axiomatic design. In the customer domain, the customer needs (CNs), or customer attributes (CAs) in Suh’s [20] terms, of the involved stakeholders are gathered [18, 21]. In the functional domain, the functional requirements (FRs) describe what the product should do. The FRs “are derived from the perceived needs of customers” [20] or, better, the perceived needs of stakeholders [21]. The realized product is based on the FRs and specified by the DPs in the physical domain [18].

Axiomatic design is based on two axioms: the “independence axiom” and the “information axiom” [18]. The independence axiom dictates that every FR is satisfied by a single DP. This process is called mapping [18]. If a DP satisfies more than a single FR, the design will be “coupled” [18]. For example, a lid on a jar not only needs to preserve the jar’s contents during transport and storage and therefore be firmly tightened, but in contrast, that same lid should also be easily opened by the users. Coupled designs can be a result of not making the correct decisions in the process of product design [20]. Meanwhile, the information axiom supposes that the design with the least information is the best. Brown [19] compares this with the amount of directions needed for a road trip. The best choice is the one with the least amount of information, as it is easiest to follow.

## Methods

**Fig. 2:**
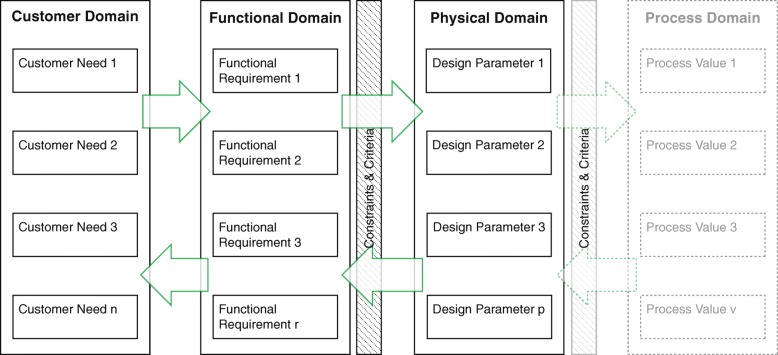
A schematic view of axiomatic design domains adapted from Suh [18] and Thompson [21]

In this research, the domains of axiomatic design are used and applied as an analyzing tool to give an overview of the mismatches in the mapping process. For this, an alternative visualization of the domains of axiomatic design is used (Fig. 2) [18, 21]. In this, the constraints and criteria are not placed underneath the FRs [21], but as a filter between FRs and DPs. For example: a criterion could be “low price.” With every translation of a FR to a DP (and Process Value), the criterion of price will be used in choosing the best alternative.

Information given by stakeholders can include CNs, FRs, DPs, and constraints and criteria; these must be sorted and categorized [19]. This can mean deducing the underlying CNs related to FRs or DPs. As different stakeholders could have different goals and wishes, the customer domain can contain conflicting needs. Choices are made during the mapping between the domains.

**Fig. 3:**
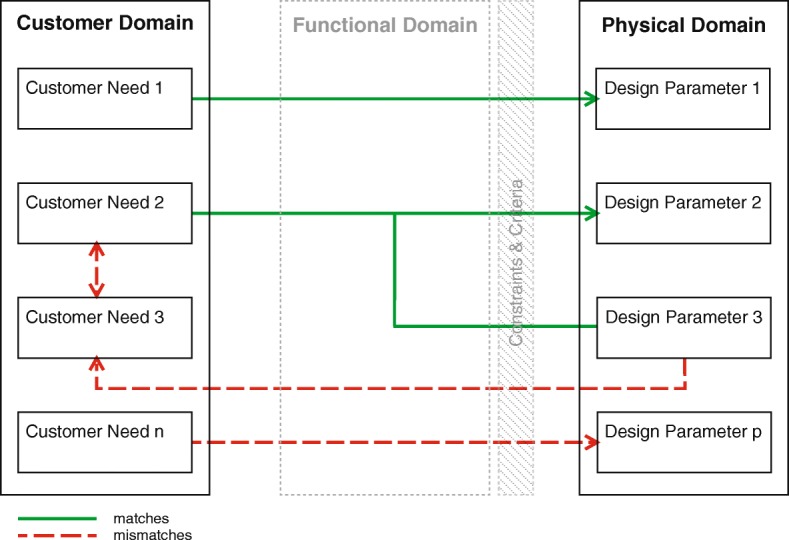
The axiomatic design method for identifying mismatches

By comparing the needs of the nurses (CNs) with the realized product (DPs), the factors contributing to the mismatch can be found. Figure 3 visualizes the principle of the comparisons, shown in the results. Matches are visualized with green continuous arrows and mismatches with red dashed arrows. The lines represent the relations between the CNs and the DPs, but cannot be considered similar to the correct mapping process.

The DPs of the MEMR are based on the software and hardware combination, as used by nurses on their units. When uncovering the CNs, the focus is primarily on the needs of the nurses. The wishes and demands of other stakeholders are also of interest as they can support or conflict with the needs of the nurses. A conflict could lead to a different DP, potentially resulting in a mismatch.

The next step is to analyze mismatches in relation to choices made in the development process. These choices influence the product quality [20]. Decisions between CNs and FRs (constraints and criteria) or the mapping between FRs and DP, with the constraints and criteria as a filter, could be the origin of part of the mismatch.

### Setting and time period

The MEMR is an in-house product developed by the IT department at a University Medical Center in The Netherlands. This academic hospital has over 20 nursing units, each of which has its own medical focus. The research was conducted on the regular nursing units. Outpatient care, medium care, and intensive care units were excluded from this research. The focus of this case study was on four nursing units. The initial research took place between December 2015 and November 2016. When an internal committee reviewed the MEMR for further development, extra interviews were conducted at the end of 2017.

### Data collection

To answer the research questions, we conducted a single case study [22] with a mixed-method design, focusing on the development process, implementation, and use of this MEMR app for nurses. A total of 16 nurses, six nurse managers, and seven other involved parties were contacted, in order to either observe their use of the app, to interview them, or for other short inquiries (see in Additional file 1: Table S1).

#### Document analysis

Using the hospital’s intranet and the internet, we searched for documents containing information on the app and its development process. An intranet search (February 9, 2016) for the name of the app returned 908 hits, of which the results of the first seven pages (sorted by “relevance”) were saved. The latter results were webpages with links to earlier finds. Later searches (April 4, 2016 and April 13, 2016) were on the working title of the app and a similar name, because documents indicated differences in spelling during the development process.

We scanned 118 items for information on the MEMR. The items were working documents, such as the list of requirements for the MEMR, Project Initiation Documents of different phases, test reports, publications in internal magazines, and videos of presentations. These documents were analyzed to establish the timeline of the product development process and to find mentions of factors relevant to the mismatch between CNs and DPs. Relevant passages of documents were copied to a database and sorted by date. The factors that influenced use were marked in this database. The timeline was used as the basis for structuring interviews and eliciting information.

#### Interviews

We conducted semi-structured and open-ended interviews with users and stakeholders, based on their involvement in the development process or use of the app. The participants were selected based on references in documents or by referral by previous interviewees. The questions for nurses were focused on expectations before use, the use itself, and their requests for improvement. Because the interviews were conducted during nurses’ shifts, some interviews were interrupted as nurses were called away. We recorded the interviewees’ responses in note form.

The interviews with nurse managers focused on their involvement in the development, testing, and implementation of the app, and on their perspectives on the acceptance of this technology by nurses. The interviews with the nurse managers of the four units were semi-structured; two were audio-recorded and transcribed, while written notes were made of the other two during the interviews.

After an initial introductory interview with the project manager of the app, we conducted a second interview using the created timeline as a basis. Subsequent follow-up questions were sent and answered by email. Also the staff member and an information manager from the department where the idea originated were interviewed. In these audio-recorded, open-ended interviews, we asked the interviewees to recount their experiences of the development and implementation processes. We also interviewed three stakeholders with commercial interests who were involved in the early development process.

#### Observation

We observed how a nurse used the app at the nursing unit where the idea of the app originated (and, indeed, where it is still used). Activities were verified according the contextual inquiry technique [23]. The patients who were measured by the nurse during the observation were informed of the research. We recorded the interviewees’ responses with written notes.

#### Use test

At the third nursing unit, where the app’s use was marginalized shortly after implementation, we examined a new implementation trial in March 2016. Notes were taken during meetings with two nurses, the nurse manager, and the project manager.

### Analysis

The analysis of the data was an iterative process that took place during the course of data collection. The working documents were created mostly by the development team. Documents on meetings and the test reports were valuable for studying the ongoing process and for identifying problems. In accordance with the principles of mixed-method research, we compared the results of the document analyses with the interviews and observations, ensuring that the data were triangulated.

We conducted the analysis via a process of factoring, which entailed finding the patterns in the collected data [24]. The factors were identified by analyzing the notes and transcripts of the interviews and (internal) documents as causes that influenced use (either positively or negatively). These were compiled in a database and compared to the findings of Gagnon [6, 7] and of Lu [5], to organize the more detailed findings. This table could be interesting for further development or implementation of the MEMR.

The specific factors leading to mismatches were identified according to the frequency with which they were mentioned and/or the importance ascribed to them by nurses and nurse managers. This is based on the number of subjects ascribing these factors in their interview, including the underlying problem or the consequences, as a barrier in use. These were placed as a top two or three per interview. Short inquiries with more specific questions were already focused on specific factors and therefore not part of this overview.

The barriers were grouped into three main focus areas on perceived impact on use, leading to the categories of mismatches. The factors were then placed in the axiomatic design domains based on the categories and traced back to decisions in the development process.

### Validity

The first author is the primary researcher in this study. During the study, the findings and reflections were discussed with the research team which also co-authored the paper. Feedback and discussion during the process of data collection and interpretation are used to refine the research method and improve the presentation of the materials.

During the use test, the findings were discussed with the project manager and the nurse manager of the unit to validate the findings. The final version of the article was sent to the project manager for an additional check of the findings relating the development.

## Results

This section outlines the timeline of the process by which the app was realized. It also specifies the involvement of the target audience. Relevant factors mentioned by nurses and nurse managers influencing the use, which can indicate a mismatch (research question 1), are analyzed and traced back to decisions in the process (research question 2).

### Development process

**Fig. 4:**
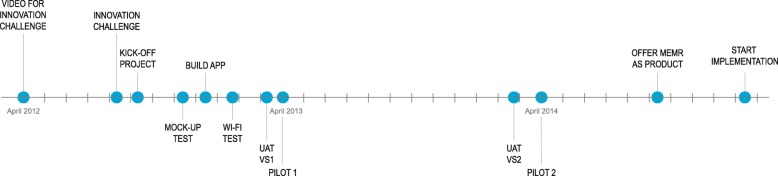
Timeline of the development process of the mobile electronic medical record

The timeline of the process is shown in Fig. 4.

A nurse and nurse manager presented the idea for the MEMR in a video [1] uploaded in April 2012 for an innovation challenge. In the video, they explain an app on a mobile phone, with which “nurse measurements immediately [are] sent to the electronic patient record after collection.” We used this and another online video [1, 25] to identify the important customer needs (CNs) of the nurses and the IT department; these are used in the schematics in the results section.

The development of this MEMR was an iterative process of designing and testing with users. A mock-up was tested for size and the quality of its user interface. The first version of the app on a small mobile device (iPod), with inputs for the five vital signs (see Additional file 2), was tested in the beginning of 2013 in User Acceptance Tests (UATs) and a first pilot on the nursing unit where the idea originated. The results were positive, even though Wi-Fi appeared to have a negative effect on the use. It was reported that there were already many requests for the app by other units.

During the next year, the development continued. Nurses were involved, including through the nurses advisory board and the nurses ICT steering group. The latter recommended the input of 12 measurements and two scores (see Additional file 2) based on commonly used functions. The UAT and the second pilot on two nursing units led to diverging results. According to research by a master’s student [26], for the first unit, which had already used the first version, there was a time gain of 88 s per patient assessment and a more complete patient record when compared to the usual paper method. For the second unit, the adoption rate dropped from 40% at the start of the pilot, to almost 10% [26]. The nurse manager attributed this to problems with Wi-Fi and limited functionality.

Before the second pilot, the IT department decided to expedite the implementation of the app in the hospital. They based this decision on the demand for the app from other nursing units and on an internal program on the utilization of tools. At the end of 2014 the IT department offered the app as a product. In the following period, it was implemented on 10 more units, using the feedback from the second pilot to improve the implementation process. The project manager indicated that if the app would be sufficiently used by nurses, it would be easier to receive a budget for further development. In the first semester of 2016, most of these units showed limited log-ins in comparison with the first unit.

### Mismatches

This section compares CNs and DPs, identifying matches and mismatches. The mismatches (also seen in Additional file 3) are explained and traced back to decisions in the development process. Corresponding FRs, derived from the internal documents, are placed in the schematics. The schematics overlap but are simplified for a clearer overview.

#### More efficient work process

**Fig. 5:**
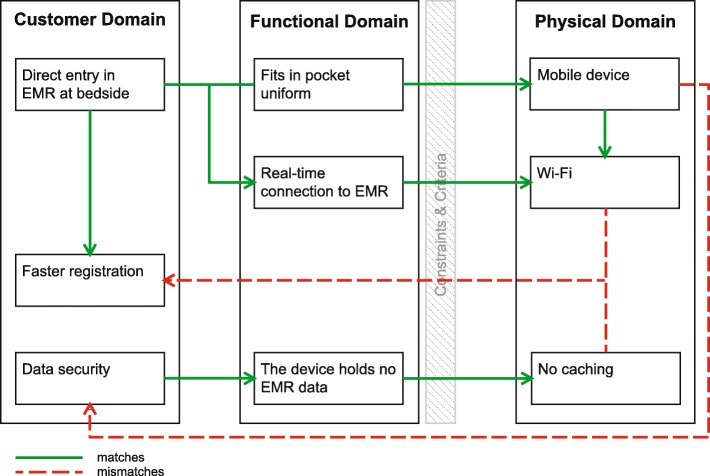
Axiomatic design schematic of efficient registration

An important CN identified by the nurses in the videos [1, 25] was a more efficient work process. They mentioned “Direct entry in the EMR at bedside” as a solution. This would eliminate the extra step of using pen and paper, which is not efficient and could lead to errors [27]. To connect the app to the EMR, the IT department chose Wi-Fi, which was available, as DP (see Fig. 5).

An important CN identified by the IT department was “data security” [5, 28]. The list of requirements submitted by the IT department stated: “the device holds no EMR data,” in case of loss or stolen devices. A document on IT architecture, concerning the quality of data, stated the importance of real-time data [29]. That document also stated limited caching as an option in case of a loss of Wi-Fi. The decision was ultimately taken not to cache patient data on the device (DP).

To use the app, a nurse needed to log-in once with full account name and password. In the nine hours after that, a fast four digit pin-code was sufficient to log-in. In blind spots (i.e. locations with no Wi-Fi) and when the devices switch between wireless access points (roaming) however, the app could lose connection with the EMR. Nurses and nurse managers reported multiple full log-ins per shift to reconnect to the database. Also, data entries were reported missing, which was attributed to “a hiccup” in Wi-Fi-connection while roaming. This conflicts with the nurses’ CN of “faster registration.”

Most interviews and several internal documents, from the test reports at the beginning of 2013 to the product and service sheets at the end of 2014, mentioned the dependency on Wi-Fi. The project manager expected that the Wi-Fi network would be optimized during the development process, so the DPs “Wi-Fi” and “no caching” for the CN “data security” of the IT department remained unchanged.

#### Functionality

**Fig. 6:**
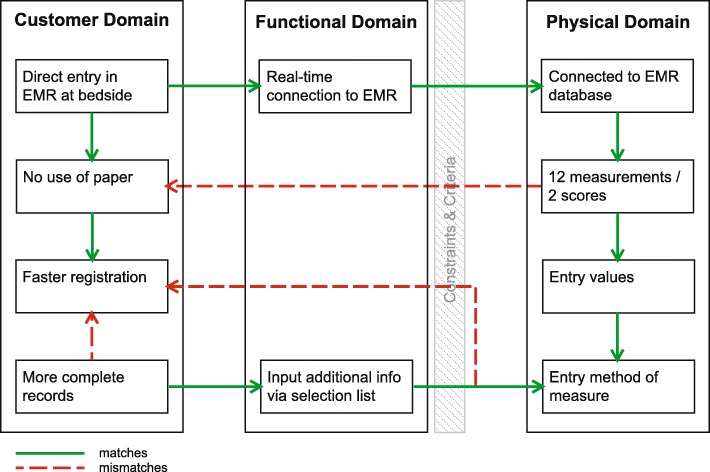
Axiomatic design schematic of functionality

The project manager indicated in the document on the overall implementation: “Experience shows that if there is functionality missing the success rate of the MEMR diminishes.” Indeed, the issue of functionality was identified as important in a number of ways. To replace paper (CN), nurses wanted to be able to enter all patient measurements in the app (see mismatch in Fig. 6). Most of the measurements could be entered using the MEMR. However, the nurses’ request for a means of registering “fluid balance” could not be met due to technical limitations. For this, the IT department was dependent on the external EMR developer. In addition, nurses mentioned the lack of other functions in the app, specific to the medical focus of their units, such as a glucose measurement function for the geriatrics unit and a Glasgow Coma Scale function for use by the neurology unit. Also, the surgical department requested the use of a camera for documenting wound healing. The IT department, however, had blocked the use of the camera on the grounds of patient privacy.

An internal newsletter reported the importance of recording measurements in the EMR, based on the demands of the Health Care Inspectorate (IGZ) and health insurance companies. It was determined during the second pilot that, compared to the traditional paper method, the first unit had more complete records [26] in less time. The app now dictated for every measurement the necessary entries, such as the value and the method of measurement. Most of this information was considered a standard mode of operation by nurses for their unit, such as the use of an ear thermometer when measuring temperature, and would not be recorded specifically. Some nurses thus perceived entering measurements in the MEMR as more labor-intensive.

#### Fit with the workflow of nurses

The need for the MEMR to suit the nurses’ workflow is a CN that was not specifically mentioned at the start by the nurses. This need became more apparent during the second pilot and the implementation. The connectivity problems influenced the workflow of nurses. A nurse mentioned assigning nurses to specific rooms. Rooms with sufficient Wi-Fi were assigned to nurses who would use the app. For rooms with limited Wi-Fi, paper could be used. Also nurses were even given the advice not to walk with the device while entering data, to avoid loss of connection with the EMR. This is contrary to the essence of a *mobile* device. A nurse manager referred to this advice: “All [given] solutions [are] likely to be the solution, but do not fit the workflow of nurses.”

**Fig. 7:**
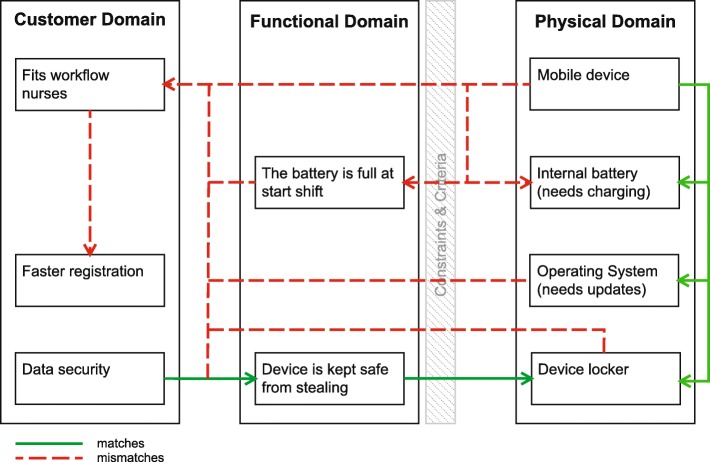
Axiomatic design schematic of fit workflow

DPs related to the supportive technology were reported as disruptive (Fig. 7). Devices with update requests, other system notifications, or errors were put back in storage, even without alerting IT support. The chosen devices had internal batteries and so were unavailable while being charged. A nine-hour battery life is needed to last a shift. In the list of requirements a comment is made on whether there was a window of three hours for charging the device. Devices that were left with empty batteries were reported to suffer from resets of actual time and date. Staff tried restoring the clock settings manually and mentioned it to be time-consuming. IT support could restore the time settings by simply connecting it with the computers connected to the app store, but these are not located on the unit.

The standard work process also influenced the acceptance and use of the MEMR. Nurses mentioned forgetting the device at the start of the shift, as it was not part of their usual routine. Visual reminders for the use were missing as at some units the devices were stored safely, but out of sight. Moreover, the task-oriented unit reported faster registration rounds (one nurse would measure all) in the second pilot, than the unit with one nurse per 3–4 patients or even one nurse per patient [26]. Note that this could also be due to a difference in the Wi-Fi quality between the units. The project manager therefore asked the nurse manager, at the start of a second implementation trial, to partly change the work method to task-oriented, to support the implementation of the app. The nurse manager refused this.

#### Other factors

During the research and second implementation attempt on the unit, other influences on the use of the app were observed and heard. An important one was hygiene, as the device was taken from patient to patient. Other mentions were the lack of clinical observation or the loss of patient eye- contact while entering data, limited functionality on the device because of data security, and a desire for extra apps on the device.

## Discussion

The importance of a continuous focus on users and of involving them in the development process, as well as the importance of tests during the iterative process, is known in (medical) product development [11, 30–35]. This is seen during the development of this MEMR, as individual nurses and nurse groups were actively involved during development and testing. The main question in this research is why the use of this app, with the involvement of nurses during the development process, decreased strongly on most units after a short use. The research questions are:

1. What were the factors contributing to this mismatch between nurses’ needs and the realized MEMR app?
2. At what point in the development process did this mismatch occur?

### Mismatches

The mismatches of nurses focus on factors in three main categories: suboptimal supportive technology; limited functionality; and limited fit with workflow.

#### Suboptimal supportive technology

The MEMR is more than just the app; the total system is composed of the app, the device, and the connection with the EMR. The reliability of the system is important for the MEMR to be adopted, as Gagnon [6, 7] also mentions. The unsuccessful implementation emphasizes Lu’s statement of the importance of wireless transmission for PDA adoption [5]. The sub-optimal Wi-Fi in combination with the decision to avoid caching was the biggest obstacle for the use of this app on most units.

The chosen DPs should be reconsidered. This could mean other solutions for the CNs. The CN “direct entry at bedside” can also be realized by using mobile computers on wheels (COWs), (touch) screens at the patients’ beds, newly developed measuring devices and wearable technologies with a connection to the EMR, or other wireless communication protocols. These other solutions are seen as competing technology by Lu [5]. The solution for the CN “data security” can also be found in allowing limited caching with encryption.

#### Limited functionality

Further development of the app could have bridged most of the mismatches between DPs and CNs in terms of functionality. The app can be made more flexible regarding the choice of measurements and default measurement methods offered to the different units. The capabilities of the device could be better utilized. For now, when it comes to functionality, the traditional paper method is still a competitor. This app is a digital translation of existing analog methods, albeit lacking the flexibility, relative swiftness, and ease of using pen and paper [27].

#### Limited fit with workflow

Kuo [15] claims that nurses should be informed that new technology is not going to alter the way of work. In this case, however, the new technology does have an influence on the workflow, although apparently there is a limit to what is accepted by nurses. The change to task-oriented work, to support technology, crossed this limit. Holden [14] also mentions the importance of compatibility with the work process for successful IT design. To support implementation and use, part of the workflow should be redesigned as well, staying close to the natural workflow of the nurses on different units [3]. This might entail, for example, enabling faster and easier reporting of problems, and supporting the new routine with visible nudges for reminding the nurses to use it and charge the battery after use.

### Development process

The origins of the mismatches are seen in the decisions between CNs and DPs, as described in the results. The effects of these combined mismatches on the expected use after implementation could be predicted by the results of the second pilot, especially by the decrease in use on the second unit. The IT department, however, had already decided that the app should be offered as a product in the hospital, based on demand. Nursing units did acquire the app, largely on the basis of the idea behind the app as seen in the CNs, as they were not able to assess the consequences of these mismatches on use. The limited use after implementation, mainly attributed to the suboptimal Wi-Fi, provided no justification for allocating an extra budget for further development. If the product is to be improved in the future, further development can identify more CNs of nurses or other stakeholders by analyzing the other influences on use.

In focusing on the factors that influence the use, we see many factors related to ICT, as Gagnon [6] named them. The technical factors are disrupting the workflow, so there is overlap. Developers should translate the mentioned DPs back to FRs and find more unmentioned CNs [36, 37]. The optional DPs should be communicated with the nurses and nurse managers, not just as the specifications. In cooperation with the nurses, the effects of the DPs on the workflow should be assessed, and the outcome should be used in the choices for DPs and even the decision on implementation.

### Use of axiomatic design

The principles of many models, such as the abovementioned UTAUT and TAM [13, 14, 16, 17], can be recognized in the results. The use of axiomatic design as an evaluation tool gives a clear and structured overview of what the nurses want and of the conflicting needs of other stakeholders. It effectively visualizes the factors contributing to mismatches between CNs and chosen DPs.

In axiomatic design, the first axiom states that one DP must only satisfy one FR and not affect others [18]. The DP of a mobile device makes it possible to enter measurements at the patient’s bedside, which allows faster registration, but it also affects the security of data. As a mobile device is easy to walk with while at work, it is also easily taken by unauthorized people. The choice then for not allowing to cache of data, resulted in missing entries, conflicts with the faster registration. This complex web of conflicting solutions shows that the product also does not satisfy the second axiom, which states that every FR is satisfied by a single DP.

### Limitations

Although the method of implementation of the MEMR was also studied to get a total overview, the focus of this research was primarily on the development process. The positive effects of active involvement or pressure by the nurse manager cannot measure up to the abovementioned barriers. The effects cannot as such be isolated.

In addition, since the second pilot and because of limited use on most units, the less mentioned factors were not researched in depth. Individual user characteristics such as age and technology-readiness are known to influence use [5–7, 17]. Hygiene, for example, is also recognized as a concern by Bauldoff [38], who has researched the introduction of mobile devices for information reference in nurse education. Also, the app as a barrier in nurse-patient contact is also mentioned by Junglas [3]. The effect of these could have been be more influential on the adoption and use than is observed in this research.

Furthermore, interviews with nurses were conducted during their work hours and were thus regularly interrupted by questions, phone calls, and exchanges of information on patients. Although nurses were willing to give feedback, care-related tasks were prioritized, so some interviews were limited in time. Despite all the available information on the intranet and the interviews conducted with involved parties, still some information may not have been uncovered.

Finally, the framework of axiomatic design was not used by the developers. The FRs mentioned in the schematics are not real functions as should be used in axiomatic design. These items were chosen as close to the descriptions used in the documents as possible. These documents mainly contained criteria, constraints, specifications (DPs), and some functions (FRs). The schematics were simplified and show only the relevant connections for that section of the research, to avoid a cluttered overview. It is possible to analyze this MEMR more in depth with the axioms of axiomatic design. This would allow for a more complex analysis but would not ensure a more detailed conclusion on the process as there is not enough information on all the choices made between the customer domain and functional domain during the development.

### Validity

At the start of the research, the first researcher was biased, based on the first interviews with a nurse manager and external parties which were the starting point of the study. The impression was that nurses weren’t involved in the development process and the usability of the app was low. Due to the methodology of the study in which data were collected from different sources, this bias did not influence the results because other sources contradicted and changed the initial impression of the first researcher. The data showed that the situation was more complex, with more factors influencing the use of the app.

### Future research

During the research, we observed that the involvement of nurses varied based on time of day, method of approach, nature of research, interest in subject, etc. This was a challenge during this study, but also is an issue during development of products and services. In our case we even had the advantage of doing a study from within the hospital, with easy access to the nursing units. The question it raises, is what would be effective approaches to eliciting responses by nurses on subjects for research or development, within their limited time and within their workflow?

It also became clear that there was a great diversity of nurses and units in the case study hospital, which caused a diversity in needs for this MEMR. As Gould [11] stresses that understanding users entails more than merely describing them, the question remains; how can this variety of users be adequately captured for internal and external stakeholders, for example designers and managers, to be valuable as inspiration, for understanding this group and for validation of concepts?

## Conclusions

With the growing shortage of nurses, one solution to limit work pressure is making the work process of nurses more efficient. During the development of supportive technology, the focus should be on the customer needs of these nurses. Extensive involvement of the target group is therefore important. This study shows that translating the customer needs of all stakeholders into design parameters that still fit the needs of nurses is essential for the adoption of this technology.

In this project, the development of this MEMR should have been ongoing, as feedback before and after the implementation showed mismatches with design parameters. These flaws could have been revised by taking a step back to focus again on the customers’ needs, to make this app support the nurses and make their task more efficient.

## Additional files

Additional file 1: **Table S1.** List of interviewed subjects and methods. (XLSX 10 kb)

Additional file 2: Overview of functionality of the two MEMR versions. (PDF 91 kb)

Additional file 3: **Table S2.** Comparison of the factors of the MEMR with the factors found by Gagnon [6, 7], Lu [5], and Bauldoff [38]. (XLSX 20 kb)

## Abbreviations

app: application
CN: Customer need
DP: Design parameter
EMR: Electronic medical record
FR: Functional requirement
MEMR: Mobile electronic medical record
UAT: User acceptance test
