# Supplementary material for: Development process of a mobile electronic medical record for nurses: a single case study
Source: BMC Med Inform Decis Mak. 2019 Jan 14;19:11. doi: 10.1186/s12911-018-0726-3 (PMC6332569; doi:10.1186/s12911-018-0726-3)
Supplement: Supplementary file 2 — Overview of functionality of the two MEMR versions. (PDF 91 kb) [file 12911_2018_726_MOESM2_ESM.pdf]

**Functionality MEMR**

**Measurements**

1. Heart Rate
2. Blood pressure
3. Temperature
4. Respiratory rate
5. Oxygen saturation

version 1

6. Oxygen intake
7. Urine production
8. Defecation
9. Weight
10. Length
11. Thorax drain fluid
12. Thorax drain air leak

**Scores**

1. Early Warning Score
2. Pain Score

version 2
